# Investigation of Roles of *TaTALE* Genes during Development and Stress Response in Bread Wheat

**DOI:** 10.3390/plants11050587

**Published:** 2022-02-22

**Authors:** Meenakshi Rathour, Shumayla, Anshu Alok, Santosh Kumar Upadhyay

**Affiliations:** 1 Department of Botany, Panjab University, Chandigarh 160014, India; meenakshirathour10@gmail.com (M.R.); shumaylasaifi5@gmail.com (S.); 2 Department of Plant Pathology, University of Minnesota, Twin Cities, Saint Paul, MN 55108, USA; alok0001@umn.edu

**Keywords:** abiotic stress, bread wheat, expression, functional divergence, TaTALE

## Abstract

The *three amino acid loop extension* (*TALE*) genes of the homeobox superfamily are responsible for numerous biological functions in plants. Herein, we identified a total of 72 *TaTALE* genes in the allohexaploid genome of bread wheat (*Triticum aestivum* L.) and performed a comprehensive investigation for gene and protein structural properties, phylogeny, expression patterns, and multilevel gene regulations. The identified TaTALE proteins were further classified into two groups, TaBLHs and TaKNOXs, which were tightly clustered into the phylogeny. The negative Ka/Ks ratio of duplicated genes suggested purifying selection pressure with confined functional divergence. Various signature domains and motifs were found conserved in both groups of proteins. The occurrence of diverse *cis*-regulatory elements and modulated expression during various developmental stages and in the presence of abiotic (heat, drought, salt) and two different fungal stresses suggested their roles in development and stress response, as well. The interaction of TaTALEs with the miRNAs and other development-related homeobox proteins also suggested their roles in growth and development and stress response. The present study revealed several important aspects of TaTALEs that will be useful in further functional validation of these genes in future studies.

## 1. Introduction

The growth and developmental processes of an organism are directed by a group of genes, known as homeobox genes. These genes encode 60 amino acids residues (AA) long homeodomain (HD) that consists of three helical structures. The first two helices are connected by a loop, while the helix-turn-helix structure is formed between the second and third helices [1]. The first plant homeobox genes, known as knotted-like homeobox, were discovered in maize [2]. The HD has been further categorized into typical and atypical based on the number of AAs. A typical HD comprised 60 AA residues, while atypical HDs consisted of more than 60 AA residues in the HD domain. For example, the three amino acid loop extension (TALE) superclass of atypical HD has three extra AAs between the first and second helices [3]. TALE superclass has been further classified into two classes; BEL-like homeobox (BLH) and knotted-like homeobox (KNOX) in plants.

The proline-tyrosine-proline (PYP) is the three amino acid loop extension that exists between helices first and second of the TALE-HD. Both the protein subfamilies KNOX and BLH of the TALE superclass contain various domains and motifs, which are important for the heterodimerization of these proteins [4]. Such protein interactions play a key role in nuclear localization and during binding affinity to DNA [5]. Besides HD, BEL1-like proteins contain highly conserved regions such as the SKY-box located in the N-terminus, the BELL domain, and the VSLTLGL motif at the C-terminal end, where bipartite domain (BELL and SKY domains collectively) also called the MEINOX interacting domain (MID) [4,6]. However, KNOX1, KNOX2, ELK, and HDs are the four characteristic domains of KNOX genes [7,8]. Later on, KNOX genes that contain the KNOX1 and KNOX2 domains but lack the ELK and HDs were recognized as the KNATM genes [9]. As per the previous findings, KNOX-BLH proteins interact to form heterodimers resulting from the interaction of the KNAT, MEINOX, and BLH MID domains [4,6]. A previous study revealed that the BLH6 and KNAT7 form a functional heterodimer that repressed the secondary cell wall biosynthesis in interfascicular fibers (IF) [10]. Many maize DNA interactions have enhanced the specificity through BEL/KNOX tandem complexes. In potato, Gel-shift assays with native DNA sequences were performed and found that binding of BEL/KNOX complexes was comparatively stronger than either protein alone [11]. TALE genes are reported to be involved in a variety of biological activities that occur during the early stages of plant organ development. For instance, a mutant of KNOX genes in maize has resulted in aberrant leaves and flowers [12]. In contrast, Reiser et al. [13] reported a gain of function via mutant that was responsible for the change of Arabidopsis, maize, tobacco, and tomato plant morphology. Similarly, KNOX proteins were discovered to be involved in meristem growth in potato and tobacco by negatively regulating gibberellin (GA) production. KNOX protein was shown to be upregulated in root nodules of soybean, whereas *SH5 BEL1*-type gene was found to be involved in seed cracking via abscission zone formation and lignin biosynthesis suppression [14].

The KNOX-BELL protein interaction can modulate hormone homeostasis [15]. These genes have been widely researched in cotton, and it has been recently discovered that the *TALE* genes contribute to the regulation of secondary cell wall production in cotton fiber [16]. Furthermore, research has revealed that heterodimerization of these proteins not only unzipped their participation in the developmental stages of plant organs but also that they are stress responders. In Arabidopsis, for example, heterodimerization of KNAT3 and BLH1 showed enhanced ABA resistance to salt stress [5]. In Arabidopsis, ABA-responsive elements play an important role in osmotic stress tolerance [17]. *TALE* genes responded to salt stress through tissue-differential expression in another investigation in poplar [18]. Above all, research has shown that TALE family proteins play a crucial role in plant developmental processes while also maintaining hormone homeostasis under stressful environments. In a recent study, these genes were found to be involved in the regulation of cotton growth and development, primarily in the cotton fiber secondary cell wall biosynthesis network [16]. Furthermore, such genes are critical in cotton organ development and abiotic stress situations [19]. *MdKNOX* genes were shown to be involved in floral induction in apples [20]. *TALE* genes were active throughout the development and wood production in poplar, and they also responded to salt stress [18]. *GmTALE8* and *GmTALE28* in soybean were shown to be increased under saline stress and dehydration [21]. Numerous studies had been carried out to decipher the functional aspect of this gene family in the majority of plant species.

Despite its immense importance, an inclusive characterization of the TALE proteins has not been performed in an important cereal crop, i.e., bread wheat (*Triticum aestivum* L.). Therefore, the current work focused on the complete investigation of the TALE gene family in the genome of allohexaploid bread wheat, a widely consumed staple crop. In the present study, the evolutionary analysis, domain and motif analysis, expression studies during tissue development stages, as well as under abiotic and biotic stress conditions, have been carried out. Aside from that, studying the interactions of these proteins with other proteins and miRNAs has provided insight into the probable roles of these proteins. This work will provide valuable insight into the functional assessment of *TALE* gene family members in bread wheat.

## 2. Results

### 2.1. Identification, Chromosomal Localization, and Characterization of TaTALE Genes in Bread Wheat

**Figure 1:**
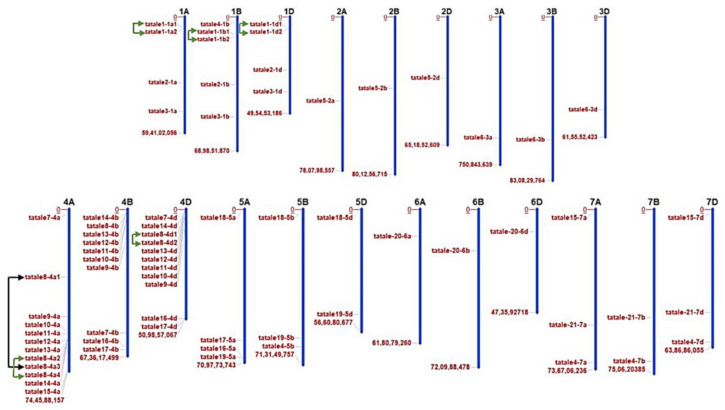
Chromosomal localization and duplication analysis. The location of *TaTALE* genes on the chromosome and subgenomes (A, B, and D) of bread wheat has been represented on the bar diagrams. The *TaTALE* genes are distributed on all the chromosomes and subgenomes of bread wheat (*T. aestivum*). Chromosome numbers are shown at the top of each bar. Black and green color curly brackets represent the existence of segmentally and tandemly duplicated genes, respectively.

The *TaTALE* genes in the genome of bread wheat (*T. aestivum*) were identified by an extensive BLAST search of the known *TALE* sequences of Arabidopsis and rice against the gene model sequences of bread wheat. The identified sequences were further searched for the occurrence of signature domains, including HD, KNOX, POX domains as described in the Materials and Methods section, which resulted in the identification of a total of 72 *TaTALE* genes (Table S1), which were named *TaTALEX-ZA*, *TaTALEX-ZB*, or *TaTALEX-ZD* where X and Z represent the gene number and chromosome number, respectively (Table S2). Based on the sequence similarity (≥90%), the *TaTALE* genes were clustered into 21 homeologous groups, and each group embodied to A, B, and D subgenomes. The genomic position information revealed the localization of *TaTALE* genes across all the 21 chromosomes of bread wheat, which varied from a minimum of 1 to a maximum of 12 *TaTALE* genes. Chromosomes 4A (12 genes), 4B (10 genes), and 4D (11 genes) consisted of the maximum number of *TaTALE* genes, followed by chromosomes 1A (4 genes), 1B (5 genes), and 1D (4 genes) (Figure 1).

The TaTALE proteins were further grouped into two classes, TaBLH and TaKNOX, having 36 TaTALE proteins in each based on the occurrence of POX and KNOX domains, respectively (Table S3). The length of TaTALE proteins ranged from 152 (TaTALE8-4A3) to 803 (TaTALE10-4D) amino acid (AA) residues with an average of 448 AA residues. The molecular weights of TaTALEs varied from 16.5 (TaTALE8-4A3) to 84.4 (TaTALE10-4D) kDa. The pI of the inferred TALE proteins ranged from 4.9 (TaTALE8-4A1) to 9.4 (TaTALE8-4B), while the predicted subcellular localization was the nucleus for all the proteins (Table S2).

### 2.2. Gene Duplication Analysis and Calculation of Non-Synonymous (Ka) and Synonymous (Ks) Substitution Rate

To relate to the *TaTALE* genes expansion and their substantial novel roles gained as a result of duplication events (DEs), the paralogous *TaTALE* genes were investigated. A total of one segmental (*TaTALE8-4A1* and *TaTALE8-4A3*) and five tandem DEs were identified. All of them belonged to the *TaKNOX* class (Figure 1, Table S3). None of the DEs was identified in *TaBLH* class. Moreover, we calculated the approximate time of duplication and scrutinized the post-duplication selection process by estimating the non-synonymous (Ka) and synonymous (Ks) substitution ratios. Analysis disclosed that all the Ka/Ks values were under 1, indicating that a robust purifying selection pressure persuaded the duplicated gene pairs with confined functional divergence. In addition, the time of divergence of the concerned gene pairs was estimated between 0.3 and 19 Mya (Table 1).

### 2.3. Phylogenetic Analysis

**Figure 2:**
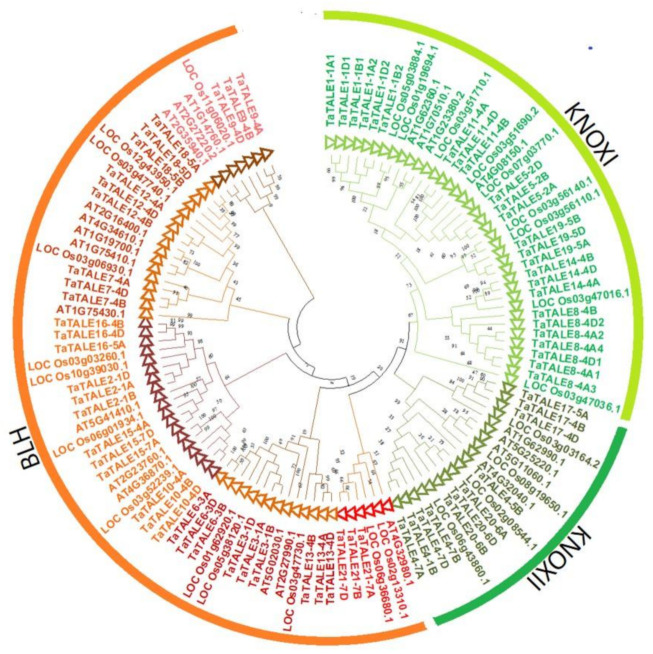
Phylogenetic analysis of the TALE proteins of Arabidopsis, rice, and bread wheat. The full-length protein sequences were used for the construction of a phylogenetic tree using the MEGAX. The tree shows two major groups, BLH and KNOX, which are further divided into subclasses highlighted with different colors.

To understand the phylogenetic relationships, the full-length sequences of TALE proteins of Arabidopsis, rice, and bread wheat were used to plot a neighbor-joining phylogenetic tree. The phylogenetic analysis revealed clustering of TALE proteins into two well-conserved classes; KNOX and BLH (Figure 2). Based on the tight clustering of wheat, Arabidopsis, and rice sequences, the KNOX class proteins were further divided into two subclasses, KNOXI and KNOXII, while the BLH clade was further divided into five (I–V) subclades. The occurrence of TALE proteins of all three plant species in the majority of subclades indicated that they appeared before the monocot-dicot split. Moreover, subclade IV consisted of TALE proteins of rice and wheat only, indicating gene loss in Arabidopsis over a long evolutionary period. All the identified homeologous TaTALEs of bread wheat were clustered in proximity, which further confirmed the high homology among them. For instance, TaTALE3-1A, TaTALE3-1B, and TaTALE3-1D homeologous proteins were tightly clustered in BLH subclade 1. Further, the paralogous TaTALEs originated by DEs were also found tightly clustered in KNOXI and KNOXII clades. For instance, *TaTALE1-1A1*, *TaTALE1-1A2*, *TaTALE1-1B1*, *TaTALE1-1B2*, *TaTALE1-1D1*, and *TaTALE1-1D2* genes were closely clustered in KNOX1 class. The results indicated the conserved function of homeologous TaTALE proteins. Further, the close clustering of paralogous TaTALEs suggested their conserved nature during the course of evolution, which was in agreement with the above results of purifying selection.

### 2.4. Exon-Intron, Domain, and Motif Analysis

**Figure 3:**
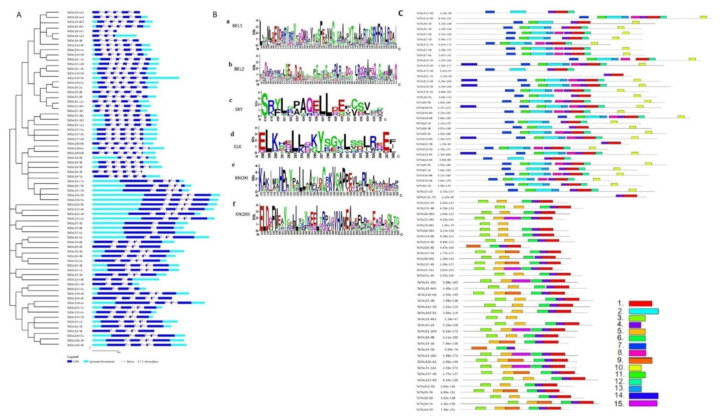
Exon-intron, domain, and motif analysis. (**A**) Exon-intron organization of *TaTALE* genes generated using the GSDS 2.0. server. (**B**) WebLogo analysis of conserved domains; (**a**) BELL1, (**b**) BELL2, (**c**) SKY in TaBLH class, and (**d**) ELK, (**e**) KNOXI, and (**f**) KNOXII of the TaKNOX class of TaTALE proteins. (**C**) Distribution of 15 conserved motifs in TaTALE proteins constructed by the MEME suite.

To gain insight into the gene structural divergence, the exon-intron analysis of *TaTALE* genes was performed (Figure 3A, Table S2). The majority of *TaTALEs* (30 genes) consisted of five exons and four introns, which was followed by six exons and five introns (20 genes). In the *TaBLH* and *TaKNOX* classes, the number of introns ranged from 2 to 6 and 2 to 7, respectively. A total of 5 and 15 *TaTALE* genes consisted of 2 and 3 introns, respectively. However, six and seven introns were present in only *TaTALE12-4B* and *TaTALE18-5B*, respectively (Figure 3A, Table S2).

The domain analysis using the SMART search and Pfam and CDD BLAST revealed the presence of conserved atypical homeodomain (HD) of 63 AA residue long stretch with the three AA loop extension between the helix one and two. Moreover, the TaBLH and TaKNOX classes of TaTALE consisted of POX and KNOX domains as their signature domain, respectively (Table S4). The POX domain of TaBLH is comprised of BEL1 and BEL2 subdomains, while the TaKNOX consisted of KNOX1, KNOX2, and ELK domains upstream to the HD. Further, the TaBLH also consisted of conserved SKY and ZIBEL (VSLTLGL) domains (Figure 3B).

A total of 15 conserved motifs were analyzed in TaTALEs using the MEME suite. (Figure 3C). Out of 15 motifs, motifs 4 and 1 were predominantly present in all the sequences. They correspond to the conserved helix 1 and helix 2 and 3 of HD, respectively. The arrangement of other motifs was according to the TaBLH and TaKNOX classes. For example, motifs 2 and 13 specifically belonged to BEL1 and BEL2 domains lying upstream of HD. Similarly, in the KNOX subfamily, KNOXI and KNOXII domains were represented by motifs 3 and 5, respectively. The SKY and ZIBEL domains, solely part of the TaBLH, were represented by motifs 7 and 10, respectively (Figure 3C). The position of domains and motifs obtained in the TaTALE proteins were highly conserved during the period of evolution, which may be correlated to their conserved functions.

### 2.5. Promoter Analysis

**Figure 4:**
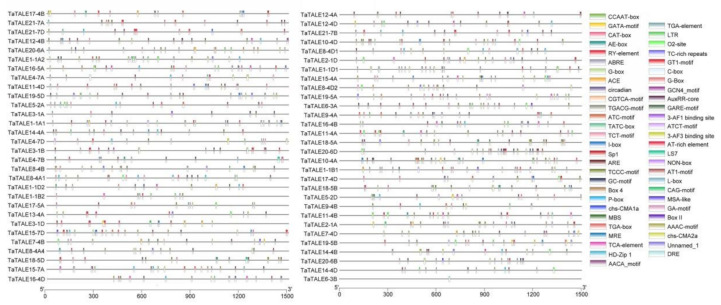
Promoter analysis *TaTALE* genes of bread wheat. TBTool was used to show the *cis*-regulatory elements represented with different colors.

At the transcriptional level, after binding to the specific DNA sequences, transcription factors control the expression of genes [22]. Thus, prediction of the DNA-binding motifs of the transcription factors is a significant approach to analyze the functional aspect of these transcription factors. Promoter regions of genomic sequences exhibit *cis*-regulatory elements (CREs) to which transcription factors bind and then mediate the gene expression [23]. Promoter regions (1500 bp upstream from the translation start site) of each *TaTALE* gene were analyzed for CREs using the PlantCare database. The study revealed a total of 54 CREs in *TaTALEs* and their length ranged from 5 to 13 bp (Figure 4, Table S5). Out of total CREs, anaerobic induction, anoxic-specific inducibility, low-temperature responsiveness, drought-inducibility, dehydration, and defense-associated CREs were found as stress-related CREs (regarded as ARE-, GC-motif-, LTR-, MBS-, DRE-, and TC-rich repeats, respectively). GATA-motif, ATCT-motif, GT1-motif, G-Box, Sp1, TCCC-motif, GT1-motif, Box 4, ACE, TCT-motif, AAAC-motif, MRE, GATA-motif, 3-AF1 binding site, AE-box, AT1-motif, ATC-motif, Box II, CAG-motif, C-box, chs-CMA1a, chs-CMA2a, I-box were identified as light related CREs. CREs, corresponding to hormonal regulation, were TGA-box and AuxRR-core (auxin related), GARE-motif, P-box, and TATC-box (gibberellin related) O2-site (zein metabolism), TCA-element (salicylic acid).

Growth- and development-specific CREs were AACA_motif (endosperm-specific negative expression), GCN4_motif (endosperm expression), HD-Zip 1 (element involved in differentiation of the palisade mesophyll cells), MSA-like (cell cycle regulation), NON-box (cis-acting regulatory element related to meristem-specific activation), CAT-box (cis-acting regulatory element related to meristem expression), RY-element (cis-acting regulatory element involved in seed-specific regulation), MBSI (flavonoid biosynthetic gene regulation), TGACG-motif (MeJA). (Figure 4, Table S5). The occurrence of an assorted range of CREs in various *TaTALE* genes suggested the diverse functions of these genes, including growth and development to the stress response.

### 2.6. Expression Analysis

#### 2.6.1. Expression Profile of *TaTALE* Genes under Tissue Developmental Stages

**Figure 5:**
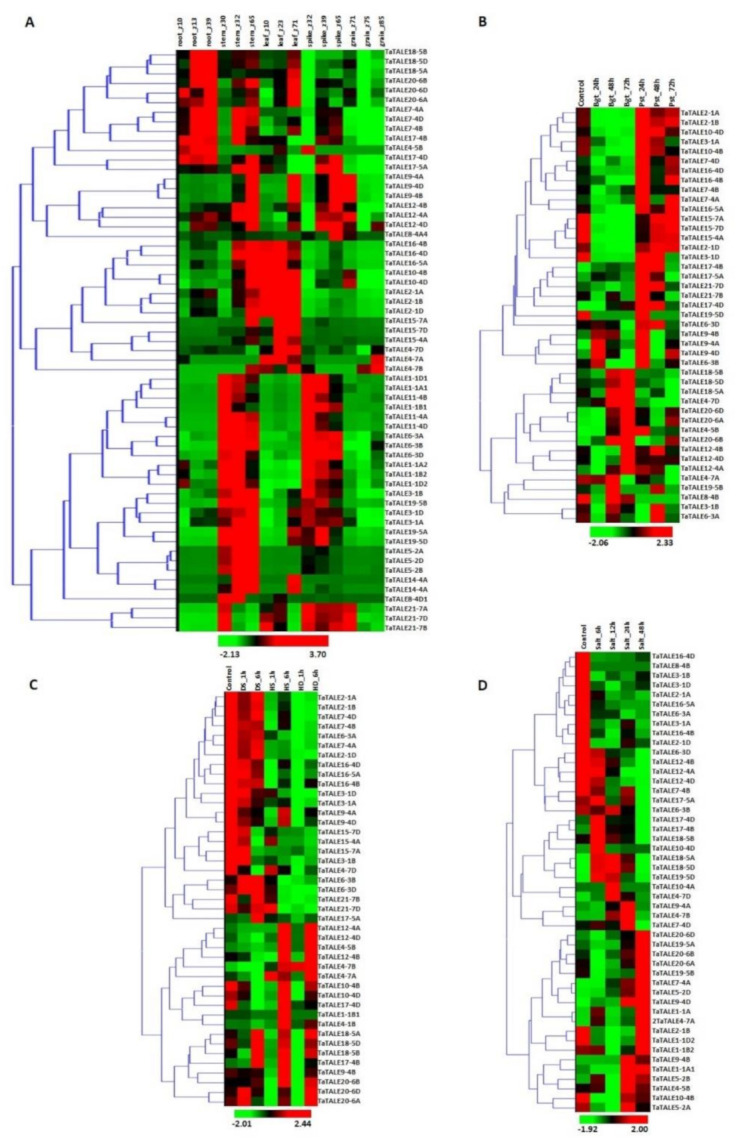
Expression analysis of *TaTALE* genes. The figure shows the expression profiling of *TaTALE* genes in (**A**) various tissue developmental stages represented by the Zadoks scale, (**B**) under biotic stress, (**C**) under heat (HS) drought (DS) and their combination (HD) stresses, and (**D**) under salt stress conditions.

The tissue-specific expression profiling of *TaTALE* genes was analyzed, using the RNA-seq derived from the URGI database comprising 15 RNA-seq libraries (Table S6), viz., root_z10, root_z13, root_z39, stem_z30, stem_z32, stem_z65, leaf_z10, leaf_z23, leaf_z71, spike_z32, spike_z39, spike_z65, grain_z71, grain_z75, grain_z85. To display the expression profile of the *TALE* genes, a heat map was created with a hierarchical clustering explorer (Figure 5A). The variable expression of *TaTALE* genes was observed in different tissue developmental stages. We observed significant expression of several genes in both vegetative and reproductive tissues. The majority of genes were found to be specific to a particular tissue or developmental stages, while some of them were expressed in more than one tissue. For instance, *TaTALE18*, *TaTALE20*, and *TaTALE7* group genes were highly expressed in all the developmental stages of root tissue and a few developmental stages of stem and leaf tissue. However, *TaTALE16*, *TaTALE15*, and *TaTALE2* group genes were highly expressed in leaf developmental stages. *TaTALE1*, *TaTALE6*, and *TaTALE19* group genes were highly expressed in stem and spike tissues, which suggested their role in both vegetative and reproductive development. Specifically, the high expression of *TaTALE7* group genes in the later developmental stages of grain suggested their role in grain filling and maturation.

#### 2.6.2. Expression Analysis under Fungal Pathogens Stress

To decipher the expression under biotic stress, the transcriptome data generated after post-inoculation with *Puccinia striiformis* f. sp. *tritici* (Pst) and *Blumeria graminis* f. sp. *tritici* (Bgt) were used (Figure 5B, Table S6). The *TaTALE* genes, *TaTALE4-5B*, *TaTALE17-5A*, *TaTALE17-4B*, and *TaTALE17-4D* belonging to the TaKNOX class, and *TaTALE9-4A*, *TaTALE21-7B*, and *TaTALE21-7D* genes of TaBLH class were found to be upregulated under exposure to Pst at 24 h; however, *TaTALE17-4D* and *TaTALE21-7D* extended up to 48 h. The homologous *TaTALE20* group genes and *TaTALE4-7A* and *TaTALE18-5B* were upregulated at 72 h of exposure to Bgt infestation, while *TaTALE4-7A* and *TaTALE4-5B* genes were highly expressed at 48 h also (Figure 5B). The homologous *TaTALE2* and *TaTALE15* group genes, as well as *TaTALE21-7D*, were found to be downregulated at 24 h, while *TaTALE4-7A* and *TaTALE4-7D* were downregulated at 48 h of *Pst* infection. The results revealed that this class of genes might play a significant and coordinated role in managing biotic stress conditions. The expression pattern also indicates that genes could be selective for the specific kind of infection and duration of infestation.

#### 2.6.3. Expression Analysis under Heat, Drought, and Combined Stress Conditions

Under conditions of high temperature, 15 *TaTALE* genes showed higher transcript levels, of which most were elevated at 6 h of heat stress, and heat-drought (HD) combined stress, which suggested the late responsive nature of these genes. In the case of drought stress, the majority of genes were downregulated except *TaTALE18* and *TaTALE 20* group genes (Figure 5C, Table S6). Altogether, most of the genes responded differentially to these stress treatments; however, some genes were hyper-responsive against specific stress treatment. Approximately 53% of *TaTALE* genes showed downregulation after HS1 and 6 h, HD 1 and 6 h in comparison to control.

#### 2.6.4. Expression Analysis under Salt Stress

A comprehensive expression profiling of *TaTALE* genes under salinity stress was carried out through transcriptome data generated from root tissue treated with 150 mM NaCl (Figure 5D, Table S6). The heatmap indicated that almost half of the *TaTALE* genes were downregulated while another half were upregulated at various hours of salt stress treatment. For instance, *TaTALE7-4A*, *TaTALE9-4B* and *TaTALE9-4D*, *TaTALE10-4D*, and *TaTALE19-5A* were elevated at the later stages (24 and 48 h) of salt stress. However, *TaTALE10-4D* was 23-fold upregulated at 6 h of salt treatment. The expression of *TaTALE17-4B*, *TaTALE17-4D*, and *TaTALE19-5D* was induced at 6 h only. In contrast, expressions of six other *TaTALE* genes tended downregulation at 6 and 12 h of NaCl treatment.

### 2.7. Interactome Analysis

**Figure 6:**
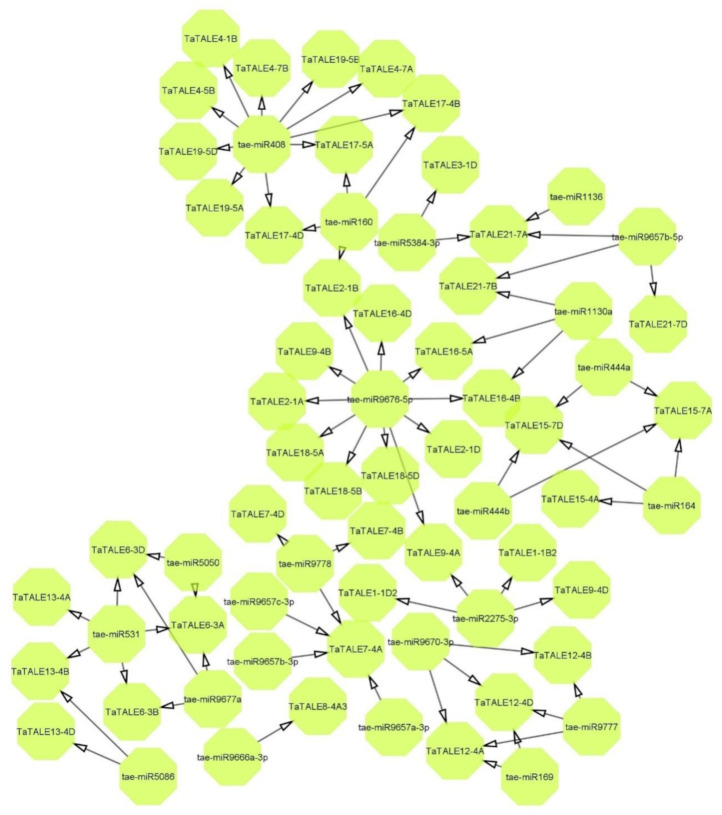
The interaction network of known miRNA of bread wheat (*T. aestivum*) with *TaTALE* genes. The prediction and generation of the network were performed by the psRNAtarget tool and Cytoscape software, respectively.

Several conserved miRNAs have been reported to be essential for reproductive development in plants, for instance, miR156/7, miR159, miR160, miR164, miR165/166, miR167, miR169, miR172, miR319, and miR396 [24]. In the present study, 30 *TaTALE* transcripts showed interaction with 23 miRNAs (Figure 6, Table S7). One miRNA such as tae-miR9676-5p was found to be interacting with 12 *TaTALE* transcripts, namely *TaTALE9-4D*, *TaTALE16-5A*, *TaTALE16-4D*, *TaTALE9-4B*, *TaTALE9-4A*, *TaTALE18-5B*, etc. In addition, tae-miR408 was found to be interacting with 10 transcripts such as *TaTALE4-7A*, *TaTALE4-7B*, *TaTALE4-1B*, *TaTALE17-4B*, *TaTALE17-5A*, etc. A majority of miRNAs were found to be interacting with one, two, or three *TaTALE* transcripts; for instance, tae-miR1130a targeted three *TaTALE* transcripts *TaTALE21-7B*, *TaTALE16-4B*, and *TaTALE16-5A*; tae-miR1136 targeted *TaTALE21-7A*, etc. Moreover, five miRNAs such as tae-miR169, tae-miR5384-3p, tae-miR160, tae-miR164, and tae-miR5086 acted as translational inhibition of their target *TaTALE* transcripts, while the other miRNAs acted by cleaving their targeted transcripts.

**Figure 7:**
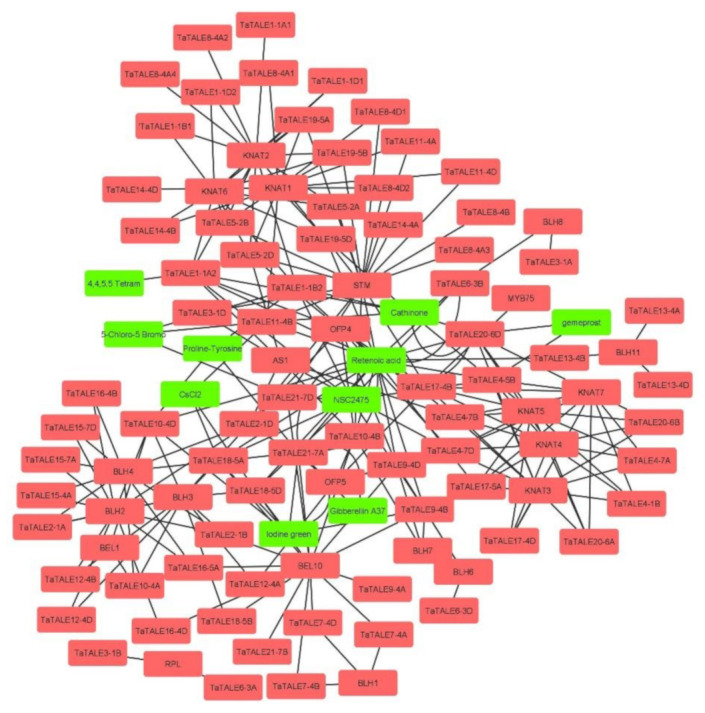
Interaction analysis of TaTALE proteins using the STRING and STITCH servers. The proteins are marked with pink color and metabolites are marked with green color. The interaction network was developed using the Cytoscape software.

Further, to identify the putative interacting proteins, the interaction network analysis of TaTALEs was performed using STRING and STITCH servers (Figure 7, Table S8). The interaction of TaTALE proteins showed interaction with 10 metabolites, which include retinoic acid, gibberellin A37, CsCl_2_ gemeprost, proline-tyrosine, 5-Chloro-5-Bromo, NSC2475, iodine green, cathinone, 4, 4, 5, 5 Tetram. For instance, TaTALE21-7A and TaTALE9-4B proteins showed interaction with gibberellin A37, TaTALE11-4B and TaTALE18-5A interacted with CsCl_2_, TaTALE20-6D and TaTALE4-5B interacted with gemeprost metabolite, etc. The TaTALE proteins also showed interactions with 23 proteins, which included AS1 (Asymmetric leaves 1), STM (shoot meristemless), homeobox protein knotted-1-like 2, BLH3, 4, 6, 7, 8, 10, 11 (BEL1-like homeodomain 3, 4, 6, 7, 8, 10, 11), RPL (REPLUMLESS), MYB75 (Transcription factor Myb75), OFP4 (Ovate family protein), KNAT1, 2, 3, 4, 5, 6, 7 (homeobox protein knotted-1-like 1, 2, 3, 4, 5, 6, 7). The majority of TaTALEs (48%) showed interaction with homeobox protein knotted-1-like protein, for instance, TaTALE11-4A, TaTALE1-1A, TaTALE17-4B, etc. followed by 45.8% and 26% TaTALEs interacted with BEL1-like homeodomain and STM, respectively. While eight, seven, six, and one TaTALEs showed interaction with OFP4, AS1, OFP5, and MYB75, respectively.

## 3. Discussion

The TALE proteins play significant roles in numerous biological functions in plants. These have been identified in numerous plants in the last few years [12,13,14,15,16,17,18,19,20,21]. However, inclusive analysis of TALE proteins in bread wheat was still lacking. Therefore, in the current study, a comprehensive characterization of TaTALEs has been performed. In the current study, we identified 72 *TaTALE* genes in the allohexaploid genome (AABBDD) of bread wheat, which was higher than Arabidopsis (22 *TALE* genes), poplar (35 *TALE* genes), *G. arboretum* (46 *TALE* genes), *G. raimondii* (48 *TALE* genes), and soybean (68 *TALE* genes) [16,18,21,25]. Polyploidy has occurred in the majority of angiosperms during the evolution, and according to an assessment of the sequenced plant polyploid genomes, the number of *TALE* genes in soybean was postulated to be connected not only to the species genome size but also to their ploidy level [21]. Similarly, the number of *TaTALE* was also higher than the other diploid genomes, which could also be directly linked to the ploidy level (i.e., allohexaploid) and genome size of bread wheat [26].

The analyses of physicochemical characteristics revealed the slightly variable molecular weight in both TaKNOX and TaBLH groups of TaTALE proteins, which is consistent with the observations in cotton [19]. TALE proteins, due to their high molecular weight, can be classified as macromolecules. The occurrence of less than 7 pI of all the TaKNOXs and the majority of TaBLHs suggested their acidic nature and a possible correlation of these proteins with secretory pathway-related proteins. The subcellular localization was predicted using the Wolfpsort (, which predicts protein localization based on multi-site, sequence homology, and functional features. The majority of TALE proteins were found in the nucleus, as expected [18,21].

The duplication events (DEs) analysis revealed the occurrence of both tandem and segmental duplications in TaKNOX class of *TaTALE* genes, which suggested the role of duplication in the expansion of the *TaTALE* gene family. Whereas, in the case of cotton (34 DEs) and soybean (91 DEs), segmental duplication events were the major cause of gene family expansion [19,21]. The gene duplication was estimated between 0.3 and 7.8 million years ago (MYA) for three paralogous pairs, while 17–19 MYA for another three pairs of paralogous genes. The results suggested that almost half of the paralogous genes were originated either before or in parallel to the hybridization event in bread wheat, while another half after the hybridization event, which occurred around 2.5–7 MYA [27]. Moreover, less than one Ka/Ks ratio of each paralogous pair of genes suggested that the genes remained fixed with their traditional functions due to the force of purifying selection [19,21].

To explore the evolutionary correlations, a phylogenetic tree was constructed, which revealed the two major groups, BLH and KNOX, and the KNOX group was further divided into KNOX I and KNOX II. A similar grouping of TALE proteins has also been reported in various earlier studies [12,13,18,21,26]. The arrangement of TALE members of different species in the same branch of the phylogenetic tree may suggest their analogous biological functions. The cladogram was distinctly divided into classes, which were further supported by our gene structure analysis. Analysis of exon-intron showed variation in the number of exons, which was consistent with the previous reports, confirming its reliability. However, the pattern of exon-intron was class-wise conserved [18,19,21]. Moreover, the *BLH* gene with seven introns is only seen in bread wheat.

The CREs mapped to the promoter sequences of *TaTALE* genes were found to be highly divergent, which further suggested their involvement in different biological processes, hormonal responses, and biotic-abiotic stresses such as anaerobic induction anoxic-specific inducibility, low-temperature responsiveness, drought-inducibility, dehydration, and defense-associated. A similar distribution of CREs has been reported in *TALE* genes of other plant species [21]. GCN4_motif (TGTGTCA) found in *TaTALE3-1B*, *TaTALE4-7A*, *TaTALE7-4D*, and *TaTALE16-4B*, is an important cis-element that plays role in an endosperm-specific gene expression. In Arabidopsis, AtPR12 with GCN4_motif was reported to be involved in protecting germinating seeds and developing seeds [28]. For all the *TaTALE* genes except the *TaTALE21* gene, we found G-Box (CACGTG) element, which has a role in response to abscisic acid, has also been reported in Arabidopsis and rice [29,30].

The analysis of the conserved domain revealed the occurrence of HD at the C-terminus of all the TaTALE proteins, which is specifically associated with DNA binding and probably involved in homodimer formation. Interestingly, the phylogenetic tree had also supported the alignment of domains and motifs in each subfamily of TaTALEs, also extended up to subclasses. The 21 AAs long ELK domain present adjacent to the HD is assumed to be responsible for the transcriptional repression; however, the exact role is still undiscovered [31,32,33]. The main function of KNOX II is homodimerization and trans-activation, while KNOX I suppresses the expression of the target genes. Moreover, the POX domain associated with the BEL1-like proteins is only found in plants and is responsible for DNA binding and protein interactions, such as in Arabidopsis with KNAT2 and KNAT5 proteins and hence play a significant role in plant development [4].

We found that *TaTALE* genes are associated with plant growth and development by acquiring insight into tissue-differential expression of these genes. Some genes were highly expressed in all five tissues, for instance, four genes of *KNOX* and six genes of the BLH subfamily. However, some were tissue-specific; for example, 16 genes showed induced expression in the development of the stem, two expressed while leaf development, and 10 were upregulated during reproductive tissues formation. These results revealed the variable role of each *TaTALE* gene, which needs to be individually validated in future studies.

The growth and development of plants highly depend upon the abiotic stresses [34]. We explored that these genes exhibited modulated expression under various abiotic stress conditions in wheat. Several hours of salt treatment increased the expression of the number of genes. The induced expression of certain genes after six hours of heat treatment further suggested the role of *TaTALE* genes in wheat against high temperatures. Under drought conditions, some of the *TaTALE* genes were highly expressed at one-hour treatment. Our investigation for biotic stress suggests that genes of both TaKNOX and TaBLH classes had shown their differential expression depending upon the type of infestation and its duration, which requires further confirmation. This information about expression patterns would support future research to ascertain the role of these genes in stress management and growth and development.

Non-coding RNAs that are endogenous and 21 to 24 nt-long are referred to as microRNAs (miRNAs). They extensively regulate growth, development, and adaptive response against abiotic stresses through controlling target genes either at the posttranscriptional or translation level of protein synthesis [35]. Through this report, we recognized miRNAs and target genes to explore specific transcripts involved in the growth and development process and responses toward different stress conditions. We found that identified miRNAs are mostly involved in cleavage mechanisms instead of translation inhibition. One of the interacting miRNA (tae-miR408) is related to plant adaptations under Pi starvation and salt stress conditions via mediating Pi acquisition under low-Pi stress and altering the ABA signaling pathway as well as osmoprotectants biosynthesis in salt stress conditions [35]. The previous result suggested that the taemiR408 regulates the target genes through a cleavage mechanism which means the targeted genes of taemiR408 were exhibited reverse expression patterns to this miRNA; hence, transcripts were downregulated under Pi starvation and salt stress [35]. Similarly, miR164 targets the NAC transcription factor family and acts as a key regulator in diverse developmental processes, for instance, lateral root development, vegetative, floral, including embryonic development [36]. In wheat, miRNA tae-miR164 targets NAC transcription factor negatively regulates resistance against stripe rust [37]. According to another study, the miR1432 targets the calcium-transporting ATPase 9, and tae-miR9657b-5p aims at calcium-dependent protein kinase (CDPK), where Ca^2+^ was proposed to have an intermediary role at the time of plant embryogenesis [38,39,40,41]. Although the functions of miRNA have been reported earlier in respect to this superfamily, they have yet to be elucidated [42].

Furthermore, studying interaction networks contributes to a better understanding of protein biological functions and molecular processes. As a consequence, the STRING and STITCH servers were used to determine which proteins and metabolites interact with wheat TALE proteins. The TaTALE proteins exhibited interaction with the homeobox protein knotted-1-like (KNAT), which is known to have a function in meristem formation and cell maintenance in an undifferentiated and meristematic state. The KNAT proteins have been linked to a variety of morphological processes during plant development [43]. Further, the interaction of KNOX proteins with members of the BLH family was found consistent with previous research indicating that KNOX and BLH recognize and bind to each other to create the KNOX-BELL heterodimer [44]. The TaTALE proteins’ interaction with other proteins such as AS1, STM, BLH, OFP4, OFP5, and MYB75 indicated that they are associated with diverse functions in plants. According to one study in Arabidopsis, BLH and STM work synergistically to ensure proper shoot development [4]. The interaction of the TaTALE proteins with the BLH and STM suggested that they might be involved in root architecture development in the proper way. In addition, the role of TaTALE proteins in the cell differentiation of leaves could be suggested because of its interaction with the AS1 transcription factor [45]. The interaction with ovulate family proteins showed that TaTALE proteins might also function as a transcriptional suppressor, which has been associated with the reduced length of various aerial organs of plants such as the rosette leaf, hypocotyl, floral organ, silique, etc. [46]. In addition, in rice, OFP2 and KNAT7 interacted to limit secondary cell wall biosynthesis [47]. As a result of the interaction study of these TaTALE proteins, we may deduce that they may play a variety of functions in plant growth and development. Furthermore, the image shows that multiple KNOX proteins interacted with BLH proteins. Arabidopsis plants exhibited enhanced BLH1 and KNAT3 genes expression under stress conditions. The absence of nuclear export signal (NES) resulted in the retention of KNAT3 in the nucleus for a longer period of time when BLH1 levels rise. The BLH1-KNAT3 complex also promotes ABI3 (abscisic acid insensitive) production after binding to the ABI3 promoter region. ABI3 transcription factor regulates abiotic stresses such as high temperature and salt stress [48,49,50]. These reports, along with our results, suggested that the TaTALE proteins might also play a vital role in stress response. However, additional study is needed to better understand the role of TaTALE proteins in the abiotic stress response.

The TaTALE proteins also showed interaction with multiple metabolites. For instance, TaTALE21-7A and TaTALE9-4B proteins showed interaction with the gibberellin A37 hormone. These TaTALE proteins also consisted of gibberellins-responsive *cis*-regulatory elements (GARE). The results suggested the role of TaTALE proteins in gibberellin metabolism and signaling. TALE has been reported to repress the gibberellin biosynthesis for meristematic cell development in Arabidopsis [51]. The KNOX proteins and gibberellin relationship has also been reported in Arabidopsis and maize. In Arabidopsis, the KNOX proteins repress the gibberellin biosynthesis, while in maize, the Knox protein negatively regulates the catabolism of gibberellin [52,53]. The multiple TaTALE proteins showed interaction with retinoic acids such as TaTALE17-5A, TaTALE4-5B, TaTALE10-4D, etc. The member of TALE proteins named PREP gene was shown to be induced by retinoic acid in zebrafish [54]. However, no such study has been reported in plants. The TaTALEs also showed interaction with several other metabolites, including gemiprost, 5-chloro-5bromo, etc.; however, we could not find any report of such interaction in the literature. These interactions need to be validated in future studies in plants.

## 4. Materials and Methods

### 4.1. Identification and Nomenclature of TaTALE Genes

An extensive BLAST search was performed for the identification of *TALE* genes in the genome of *T. aestivum*, *A. thaliana* and *O. sativa* TALE sequences (Table S1) were used as a query against the protein model sequences of wheat downloaded from IWGSC (IWGSC RefSeq assembly v2.0). (http://wheat-urgi.versailles.inra.fr/Seq-Repository/Genes-annotations, accessed on 25 February 2019; http://www.wheatgenome.org/ accessed on 25 February 2019). The hidden Markov model (HMM) and Pfam Blast search at e-value 10^−10^ were used to search for the POX (PF07526) and KNOX (PF03790 or PF03791) domains and further confirmed by SMART and NCBI Conserved Domain Database [55,56]. The nomenclature of identified TaTALEs was performed as per the international rules for gene symbolization of *T. aestivum* (http://wheat.pw.usda.gov/ggpages/wgc/98/Intro.htm accessed on 6 October 2021).

### 4.2. Chromosomal Localization, Homeologs Prediction, and Duplication Events

The Plant Ensembl (http://plants.ensembl.org/Triticum_aestivum/ accessed on 10 August 2021) was used for predicting the chromosomal and sub-genomic localization of *TaTALE* genes. Identification of the homeologous grouping in *T. aestivum* was carried out on the basis of sequence similarity. The sequences with ≥90% similarity were considered homologous, as performed in earlier studies [57]. The chromosomal distribution of *TaTALE* genes was plotted with the MapInspect software (http://mapinspect.software.informer.com/ accessed on 1 October 2021) by mapping each *TaTALE* sequence with the respective chromosome sequence. For the prediction of duplication events, the MAFFT software was used, and the sequences with 80–90% sequence similarity were regarded as duplicated genes. Further, on the basis of the distance between them, duplicated genes were regarded as tandem and segmental duplication event [58].

### 4.3. Phylogenetic Relationship and Multiple Sequence Alignment

Multiple sequence alignment (MSA) was performed to obtain the conserved amino acid residues using the MultAlin and MUSCLE software at default parameter, and a logo was created using the WebLogo 3 software [59,60]. For the phylogenetic analysis, the full-length protein sequences of Arabidopsis, rice, and wheat were aligned by the MUSCLE program, and the tree was constructed using the neighbor-joining method through MEGAX software with bootstrap replicates set to 1000 [61].

### 4.4. Synonymous and Non-Synonymous Substitution Rates of TaTALEs

The protein and genomic sequences of the duplicated *TaTALE* genes were aligned to calculate synonymous substitution per synonymous site (Ks) and non-synonymous substitution per non-synonymous site (Ka), and Ka/Ks ratio was also computed with the help of TBtool software [62]. Further, the Ks value was used to calculate the divergence time (T) of each pair of duplicated genes using the formula T = Ks/2r, here r represents divergence rate, which was assumed as 6.5 × 10^−9^ for cereals [63].

### 4.5. Gene Structure Analysis

The gene structure was investigated in terms of exon-intron organization and intron phases by aligning the respective genomic and CDS sequences of each *TaTALE* gene. The exon-intron organization was displayed using GSDS 2.0 server [64]. Further, *cis*-regulatory elements were analyzed from the 1.5 kb upstream region of each *TaTALE* gene through the PlantCARE database [65] at default parameters and represented using TBtool software [62].

### 4.6. Physicochemical Analysis of TaTALE Proteins

The physicochemical properties such as the peptide length, molecular weight, and isoelectric point were analyzed by the ExPasy tool, which was further confirmed by the Ensemble plants database [66]. Subcellular localization was predicted by the WoLF PSORT [67]. Domain and motif analysis was scanned through the SMART server and MEME (Multiple Expectation Maximization for Motif Elicitation) suite version 5.1.1, respectively [55,68].

### 4.7. Expression Profiling under Tissue Developmental Stages and under Abiotic and Biotic Stress Conditions

Using the high-throughput RNA sequencing data (accession number ERP004714) available in two biological replicates, a genome-wide expression investigation of *TaTALE* genes in five tissues (root, stem, leaf, spike, and grain) and their three developmental stages was performed [69,70]. Using the Trinity software [71], the expression value was computed in fragments per kilobase per million reads (FPKM). The expression value of each gene was further confirmed at the Expression ATLAS [72].

To investigate the effect of abiotic stimuli, the differential expression of *TaTALE* genes under salt stress was studied. RNA-seq data generated from roots after 6, 12, 24, and 48 h of NaCl treatment were used for the expression analysis [73]. The differential expression of *TaTALE* genes in the presence of heat (HS), drought (DS), and their combination (HD) was also analyzed using the available high-throughput RNA-seq data [74]. The data were generated from leaves tissue after the treatment of HS (40 °C), DS (20% PEG), and HD stress for 1 and 6 h.

For biotic stress, the RNA-seq data generated by Zhang et al., 2014 [75] was used which was obtained in two biological replicates after the infestation of two fungal pathogens; *Blumeria graminis* f. sp. *tritici* (Bgt) and *Puccinia striiformis* f. sp. *tritici* (Pst) for 24, 48 and 72 h in seven-day-old leaves [75].

The differential expression analysis from the Trinity package in terms of FPKM was carried out using the following parameters: FDR < 0.05 and 0.001 *p*-values [71]. The heat maps were created using the Hierarchical Clustering Explorer 3.5 and following the Euclidean distance approach [76].

### 4.8. miRNA-Targets and Interaction Analysis

The targeting miRNAs for the *TaTALE* transcripts were recognized by searching the genes coding sequences against the published miRNAs in the *T. aestivum* genome through the psRNATarget database [77] and finally visualized via the Cytoscape software.

For the prediction of putative interacting protein partners, the STRING server was used (http://stringdb.org, accessed on 15 February 2022) [78]. Whereas, to study the protein chemical interaction, the STITCH server was used (http://stitch.embl.de/ accessed on 15 February 2022) [79]. The STRING network generated using TaTALE proteins was further expanded from the STRING Cytoscape App using default parameters to identify the various metabolites. The final interaction network was generated through the Cytoscape software (https://cytoscape.org/download.html, accessed on 15 February 2022).

## 5. Conclusions

In conclusion, an extensive characterization of TaTALEs has been carried out. Members of the TaTALE family were found evolutionary and functionally conserved. The modulated expression profiling of *TaTALE* genes in distinct tissues and stress conditions suggested their biological functions in growth and development and stress response, which were further verified by interaction network analysis. The interaction network analysis found that these proteins interacted with various proteins and metabolites involved in plant development and other processes, indicating that they are involved in a plethora of plant development-related activities. These genes were also shown to be controlled by miRNAs. The current analysis revealed several essential characteristics of these proteins, which need to be experimentally confirmed in detail in future investigations.

## Figures and Tables

**Table 1:** The calculation of Ka/Ks ratio and divergence time of duplicated *TaTALE* gene pairs.

Paralogous Genes		Ka	Ks	Ka_Ks	Duplication Event	T(MYA)	Selection Pressure
TaTALE8-4A3	TaTALE8-4A1	0.0244421	0.028216	0.8662338	SD	2.150647397	Purifying
TaTALE8-4A2	TaTALE8-4A4	0.0013357	0.004329	0.3085478	TD	0.329955514	Purifying
TaTALE8-4D2	TaTALE8-4D1	0.0515728	0.102674	0.5022977	TD	7.825746904	Purifying
TaTALE1-1D2	TaTALE1-1D1	0.0517904	0.25453	0.2034744	TD	19.40018629	Purifying
TaTALE1-1A2	TaTALE1-1A1	0.0572889	0.239382	0.2393203	TD	18.24556926	Purifying
TaTALE1-1B2	TaTALE1-1B1	0.0595657	0.235113	0.2533489	TD	17.92021991	Purifying

## Data Availability

All the data used in this study are freely available in the databases /repositories. Link and accession number have been mentioned in the manuscript.

## Supplementary Materials

The following supporting information can be downloaded at: https://www.mdpi.com/article/10.3390/plants11050587/s1, Table S1: List of *TALE* genes in *Arabidopsis thaliana*, *Oryza sativa*, and *Triticum aestivum*; Table S2: Analysis of various characteristic features of *TaTALE* genes and proteins of *Triticum aestivum* L.; Table S3: List of paralogous *TaTALE* genes in *Triticum aestivum* L.; Table S4: Domain organization in TaTALE proteins of *Triticum aestivum* L.; Table S5: The categorization of various *cis*-acting regulatory elements in the upstream of *TaTALE* genes; Table S6: List of high-throughput RNA-seq data used for expression profiling under tissue developmental stages and biotic and abiotic stress conditions; Table S7: List of interacting miRNA as target mimic or direct target of TaTALE genes; Table S8: List of interacting co-expressed genes of TaTALEs generated from String and Stitch servers.
